# Experimental subjects do not know what we think they know

**DOI:** 10.1038/s41598-019-57395-7

**Published:** 2020-01-24

**Authors:** Jared M. Field, Michael B. Bonsall

**Affiliations:** 1 Wolfson Centre for Mathematical Biology, Mathematical Institute, University of Oxford, Oxford, OX2 6GG United Kingdom; 2 Mathematical Ecology Research Group, Department of Zoology, University of Oxford, Oxford, OX1 3PS United Kingdom; 3 School of Mathematics and Statistics, The University of Melbourne, Parkville, 3052 Australia

**Keywords:** Behavioural ecology, Applied mathematics

## Abstract

Many biological, psychological and economic experiments have been designed where an organism or individual must choose between two options that have the same expected reward but differ in the variance of reward received. In this way, designed empirical approaches have been developed for evaluating risk preferences. Here, however, we show that if the experimental subject is inferring the reward distribution (to optimize some process), they will rarely agree in finite time that the expected rewards are equal. In turn, we argue that this makes discussions of risk preferences, and indeed the motivations of behaviour, not so simple or straightforward to interpret. We use this particular experiment to highlight the serious need to consider the frame of reference of the experimental subject in studies of behaviour.

## Introduction

While in physics it is standard, in biology we do not often think about frames of reference. Suppose, for example, an experiment is set-up such that an organism (or person) must choose between two options with the same expected reward but different variance in reward. Further, suppose that the organism consistently chooses the safe bet (lower variance). In this case, we may be tempted to label that organism as risk-averse. This makes sense from our point of view or frame of reference. However, are we sure that the organism agrees on the experimental set-up? Perhaps, from the frame of reference of the organism, the expected rewards are not even perceived to be the same. If this is the case, we ought to be careful with the conclusions we draw about why the organism is behaving a certain way. Though this particular experiment was picked to make a point, hundreds of variations on it have been carried out across several disciplines (see reviews^1–3^ and references therein).

Most theoretical attempts to understand behaviour start by assuming some sort of quantity to be optimized^4^. In finance, classically it is return on investments under a certain risk constraint^5^. In economics, it is (broadly defined) utility^6^. In biology, utility is usually replaced with reproductive values and assumptions of rationality with natural selection^7^. However, energy budgets and threshold reserves have also proved fruitful in understanding risky behaviour^8^. Here, however, we divorce ourselves of any such quantities. Instead, we shift attention to the information available in order to make decisions. This way the focus is put on potential differences in beliefs between experimenters and those on whom they are experimenting. We are interested not in predicting traits or behaviours in experiments but in the conclusions we can and cannot draw from them.

In this paper, as is standard in statistical decision theory, we assume that the organism or person being studied is sampling their environment and updating their beliefs^9–12^. In particular, in the experiment described above (and below, in more detail), we suppose that for each of the options the organism is inferring the probability of receiving a reward at any instance. Our analyses show that, given infinite time, the organism can indeed infer correctly this probability. In other words, the organism will agree with the experimenter on the experimental set-up (which is to say, agree that the expected rewards of each option are equal). However, we then go on to show that in finite time such agreement will rarely be reached. This is clearly important as all experiments, by nature, have a fixed end-point. In light of this, we point out that it is not so simple to infer risk preferences, as is often done, from these types of experiments. The problem, as we point out, arises from the experimenter and experimental subject having different frames of reference. It is worthwhile noting that the problem of finite sampling and its effect on belief dynamics has been considered in the economics literature^13–15^. There, however, the focus is on understanding the consequences of having an incorrect model of the world such as the fallacy of the Law of Small Numbers. A further difference is that we study not only the absolute belief dynamics of an agent but the deviation from a point of reference *i.e*. the beliefs of the experimenter. This is crucial to understand motivations of behaviour properly.

The rest of the paper is organized as follows: in the next section we go into more detail on the broad class of experiments we are using to demonstrate our point. Following this we introduce the inference problem and prove convergence to the true probability of receiving a given reward given infinite time. Next, noting that real experiments are finite, we show that such a convergence does not occur in this case. Further, we show that the ramification of this is that the experimenter and experimental subject do not agree in finite time on the parameters of the experiments. Otherwise put, for any finite number of trials the organism (or person) being experimented on will seldom believe that the expected rewards of each option are equal. Next, we consider a case study and provide some practical insights for experimentalists. Finally, we summarise our findings and consider their broader implications.

## Experimental Problem

To motivate our problem, we consider an experimental design where an organism is presented with two choices. One choice (called an arm) leads always (*i.e*. with probability ${\hat{p}}_{1}=1$) to a fixed reward of *c*. The other choice (the other arm) leads with probability ${\hat{p}}_{2}$ to a reward of *a* and $(1-{\hat{p}}_{2})$ to a reward of *b*. The experiment is designed such that the expected reward is the same on both arms. This leads to,

1 $$c={\hat{p}}_{2}a+(1-{\hat{p}}_{2})b.$$

In this way, from the point of view of the experimenter, if only expected values are used then the organism should be indifferent to each arm. In light of this, in order to make decisions the organism ought to consider the variances in rewards. For an excellent (and extensive) review of these types of experiments see^1^. However, as we show below, from the point of view of the organism, the expected rewards are not always equal.

## Inference Problem

Suppose an organism is attempting to infer the distribution of two possible pay-offs on an experimental arm of the type described above. In particular, by sampling the past pay-offs they are attempting to infer, in a Bayesian manner, the probability *p* of receiving *g* and the probability 1 − *p* of receiving *h*. In order to do this, both a prior distribution and likelihood function will be needed to form a posterior distribution for *p*. To this end, suppose *s* successes and *f* failures (with regards to receiving *g*) are observed. In this case, the likelihood that *p* = *x* will be given by

2 $$Pr(s,f|p=x)=(\begin{array}{c}s+f\\ s\end{array}){x}^{s}{(1-x)}^{f}.$$

To encode any previous knowledge we choose the conjugate prior of the binomial distribution, the Beta distribution, which is given by:

3 $$Pr(p=x)=\frac{{x}^{\alpha -1}{(1-x)}^{\beta -1}}{B(\alpha ,\beta )},$$

where *B*(*α*, *β*) is the Beta function with *β* > 0 and *α* > 0. The benefit of using this prior is two-fold. First, being the conjugate prior of (2), it makes possible the calculation of a closed-form posterior distribution. Second, the hyper parameters *α* and *β* allow for the encoding of an incredibly wide range of prior beliefs (for example *α* = *β* = 1/2 leads to the Jefferys prior whereas *α* = *β* = 1 leads to the uniform prior). Finally, by a straightforward application of Bayes rule, the posterior distribution is found to be given by

4 $$Pr(p=x|s,f)=\frac{{x}^{s+\alpha -1}{(1-x)}^{f+\beta -1}}{B(\alpha +s,\beta +f)},$$

which is also a Beta distribution. In this way, as the experimenter provides the organism with additional pay-offs (one way or the other) the organism can infer a distribution about *p*, the probability of receiving *g*. Note that the mean of (4) is given by

5 $$\tilde{p}=\frac{\frac{\alpha }{n}+\frac{s}{n}}{\frac{\alpha }{n}+\frac{\beta }{n}+1},$$

where *n* = *s* + *f* is the total number of trials thus far. In this way, if the true value of *p* is $\hat{p}$, then by giving the correction ratio of *s*/*n* the organism can indeed infer the true value as *n* gets large. Otherwise put

6 $${\tilde{p}}_{\infty }=\mathop{\mathrm{lim}}\limits_{n\to \infty }\frac{\frac{\alpha }{n}+\frac{s}{n}}{\frac{\alpha }{n}+\frac{\beta }{n}+1},$$

7 $$=\hat{p}.$$

Observe that the prior information contained in *α* and *β* is washed out (those terms go to zero), so if the experiment is carried out correctly and for long enough ($n\to \infty$), the prior beliefs of the organism do not matter.

## Finite Experiments

In the previous section we showed that if the experiment is carried out *ad infinitum* then the organism can make the correct inference. With fixed rewards, they will therefore agree that the expected rewards of each arm are equal. However, experiments are necessarily finite. We now consider the implications of this.

Recall that in the experiments we are considering on one arm, from the point of view of the experimenter, a pay-off of *c* is guaranteed. In other words ${\hat{p}}_{1}=1$. On the other arm, with probability ${\hat{p}}_{2}$ a reward of *a* is given and probability $(1-{\hat{p}}_{2})$ a reward of *b*. The experiment is designed such that

8 $$c={\hat{p}}_{2}a+(1-{\hat{p}}_{2})b,$$

so that, with perfect knowledge (the frame of reference of the experimenter), there should be no preference for either arm if decisions are based solely on averages.

However, from the point of view of the organism, at trial *n* the estimated ${\tilde{p}}_{1}$ (setting *s* = *n* in (5)) will be given by

9 $${\tilde{p}}_{1}=\frac{\frac{\alpha }{n}+1}{\frac{\alpha }{n}+\frac{\beta }{n}+1},$$

whereas ${\tilde{p}}_{2}$ will be estimated by

10 $${\tilde{p}}_{2}=\frac{\frac{\alpha }{n}+\frac{s}{n}}{\frac{\alpha }{n}+\frac{\beta }{n}+1},$$

assuming the same initial prior for both arms. Hence, for the organism to believe that the average pay-off on each arm is the same the following equality must hold:

11 $$c{\tilde{p}}_{1}={\tilde{p}}_{2}a+(1-{\tilde{p}}_{2})b,$$

which, using (9), (10) and simplifying is equivalent to

12 $$c=(\frac{\alpha +s}{\alpha +n})a+(\frac{\beta +n-s}{\alpha +n})b.$$

Using (8) on the left-hand side this is in turn equivalent to

13 $$({\hat{p}}_{2}-(\frac{\alpha +s}{\alpha +n}))a+(1-{\hat{p}}_{2}-(\frac{\beta +n-s}{\alpha +n}))b=0.$$

The above statement can be satisfied in two ways. First, as *a* and *b* are, by design, greater than zero the above statement is true if both cofactors are equal to zero. In particular, it must be that

14 $${\hat{p}}_{2}=\frac{\alpha +s}{\alpha +n},$$

and

15 $${\hat{p}}_{2}=\frac{\alpha -\beta +s}{\alpha +n}.$$

Note that both (14) and (15) can hold in only two ways. First, both may be true if *β* = 0. However, in this case the prior (3) is not a true distribution. When $\beta \to 0$ we can, in fact, interpret (3) as a Dirac delta function at *x* = 1. Though in this case the entire problem is trivial as we no longer have any uncertainty. Second, *n* may get arbitrarily large. However, in this section we are interested in precisely when this does not happen *i.e*. when *n* remains finite. Alternatively, (13) may hold (solving for ${\hat{p}}_{2}$) precisely when

16 $${\hat{p}}_{2}=\frac{a(\alpha +s)+b(\beta -s)-b\alpha }{(\alpha +n)(a-b)}.$$

However, in this case the problem now lies with the variances in reward amount. In particular, there is now no guarantee that either variance will be perceived to always be smaller or larger than the other. This can be seen by noting that the variance in reward on each arm can be written as ${\sigma }_{1}^{2}={\tilde{p}}_{1}(1-{\tilde{p}}_{1}){c}^{2}$ and ${\sigma }_{2}^{2}={\tilde{p}}_{2}(1-{\tilde{p}}_{2}){(a-b)}^{2}$, respectively and recalling constraint (12). Intuitively, as the inference process progresses the relative sizes of these variances may differ depending from which direction (above or below) ${\tilde{p}}_{1}$ and ${\tilde{p}}_{2}$ approach their true values ${\hat{p}}_{1}$ and ${\hat{p}}_{2}$, respectively as well as the relative sizes of *a* and *b*. In other words, in finite experiments (11) will rarely hold. In the one instance that it does, there is then potential disagreement in variances and perceived variances. The ramification of this is that even though the experimenter designs the experiment so that average pay-offs are equal, an organism that is performing these inferences will seldom agree in finite time. In this way, discussions of risk-preference to explain these broad group of experiments may be misleading.

## Case Study

In the seminal work of Caraco *et al*.^16^, the effect of ambient temperatures on risk-sensitivity is studied in yellow-eyed juncos. Here, juncos were given the option (coded by colours) between a constant number of seeds and a variable number with mean equal to the constant reward. The first 16 trials of every experiment were forced so that the birds learnt the distribution associated with each colour, before any decisions were permitted. The variable arm always gave each reward with equal probability (hence in this case *s* = 8). If we assume a given bird starts with a uniform prior (*α* = *β* = 1) so that there is no initial bias, the perceived expected rewards on the variable arm (right-hand side of (11)) can be calculated to be

17 $${\mu }_{2}=\frac{9a+9b}{18}.$$

Similarly, the perceived expected rewards on the constant arm (left-hand side of (11)) will be given by

18 $${\mu }_{1}=\frac{17c}{18},$$

so that the discrepancy of the two at the sixteenth trial will be given by

19 $$|{\mu }_{1}-{\mu }_{2}|=\frac{|17c-9a-9b|}{18}.$$

Clearly, for most values of rewards *a*, *b* and *c* (which in fact change for each bird in this study) the total expected rewards are not perceived to be equal. Importantly, however, note that the experimentalist can reduce this discrepancy not only by increasing *n* (the intuitive option), but also through a careful choice of *a*, *b* and *c*.

## Discussion

Hundreds of experiments have been designed such that given two choices the expected reward, but not the variance in reward, is equal on each choice^1–3,17,18^. This way experimenters attempt to infer risk preferences for organisms under a range of circumstances. If an organism consistently chooses the option that has less variance in reward they are deemed risk-averse. Conversely, if the organism chooses the option that has more variance they are deemed risk-prone. While risk-averseness occurs most frequently, risk-proneness has also been observed^1^. Here, however, we have shown that these labels may be misleading. More importantly still, we have highlighted the importance of frames of reference in biology. In particular, we have stressed the potential pitfalls of studying behaviour without consideration of the point of view of the experimental subject. It makes, unfortunately, little sense to say that an organism will not make decisions based on expected rewards simply because we have programmed the experiment so that expected rewards are equal; there is no guarantee that the organism perceives the rewards to be equal. Note that this arises for reasons entirely separate to those attributable to the Weber-Fechner law. As the Weber-Fechner law concerns the change in intensity of some stimulus required in order to notice a difference in the stimulus, it applies even in the absence of risk defined as variance (for a discussion of the economics/psychology split see^19^). In other words, Weber-Fechner applies to a much broader class of problems. Moreover, the problem studied here would arise even in the absence of imperfect perception (i.e. if the change in intensity required to notice a difference in some stimulus were able to get infinitesimally small). Our issue is akin to the problems encountered in anthropomorphising animal behaviour whereby our own beliefs or motivations are projected onto non-human animals^20,21^. However, in studies of humans, where we do (and indeed are able to) inform participants of probabilities, this study may have less relevance.

Bayesian approaches to understanding animal behaviour and in particular the use of statistical decision theory is of course nothing new^7,9,22^. Most studies, rightly so, have focused on predicting decisions or phenotypes. More recently, others have considered the biological value of information itself^10,11,23^. Here, however, we focus instead on the limits of what a Bayesian organism can know before making decisions in the above experimental context. It is important to note that, unlike other studies, our work is divorced of any quantities to be optimised in order to make decisions such as utilities, reproductive values or energy budgets^6,8,12,24,25^. In this way, our work is about conflicting perceptions of the experimenter and the organism and therefore any inferences the experimenter can make about the organism, no matter what currency is optimised.

In this paper, we have assumed that the organism in question is performing a Bayesian inference on the probability of receiving a certain reward. Starting with a general prior distribution, and sampling past rewards, we explicitly calculated a closed-form posterior distribution. A valid criticism, of course, is that organisms may not be behaving in a strictly Bayesian way. Indeed, much work has been done on this very question^26–28^. However, as the Bayesian solutions are the optimal ones, we should expect natural selection to have moulded organisms that at least approximate Bayesian behaviour via so-called Rules of Thumb^7,9,29^. A further criticism, which pervades all of Bayesian analysis, is our choice of prior. Again we reiterate that it makes possible the calculation of a closed-form posterior. More importantly, however, we emphasise the versatility of the Beta distribution afforded by its hyperparameters *α* and *β* which can control its concavity, skewness, symmetry and more. Further, for the particular values of *α* = *β* = 1/2 and *α* = *β* = 1 the Beta distribution reduces to the Jefferys and standard uniform distributions, respectively. We believe these two noninformative distributions are particularly important for this study as, from the beginning of the experiment, there is no reason to suspect the organism has bias towards any initial value of *p*. For an extensive discussion of Bayesian prior choice, and in particular the use of noninformative priors, see Berger^30^.

With this set-up we found that while from the frame of reference of the experimenter the two choices have the same expected reward, any Bayesian organism will seldom agree in finite time. In light of this, we perhaps cannot appeal to differences in variance of reward to explain behaviour in these experiments. We are not implying then that organisms are only using averages to make decisions as in the elegant early work of Charnov^31,32^. Instead, we are pointing out that for these broad class of experiments we may not be able to appeal to variances so strongly as an explanatory variable. Worse still, even if the expected rewards are perceived to be equal, there is no guarantee the variances will be perceived to be as designed. For this reason, we advocate for the inference of potential beliefs (both expected rewards and variances) of the experimental subject before motivations of behaviour are concluded. From a practical standpoint, by considering the seminal work of Caraco and colleagues^16^, we have also shown that the experimentalist can (and should) influence the inferred beliefs of the experimental subject not only through the number of trials but also through careful choice of the reward sizes. It is important to note that the modelling of this particular study has been carried out before. In their impressive review, Kacelnik and Bateson^1^ attempt to ascertain how many trials must be carried out before the proportion *p* can be estimated with a given certainty. This frequentist analysis however relies on the normal approximation of the binomial, which in turn relies on the central limit theorem. This approximation is well known to be particularly poor if either the true value of *p* is close to zero or unity or if sample sizes are small (which, of course, in these experiments they are: in the Caraco study training was restricted to 16 trials).

Though we have focussed on one class of experiments, our work points to the largely overlooked problem of frames of reference in studies of behaviour. If there is a mismatch between the beliefs of the experimenter and experimental subject, then we must be cautious to not draw conclusions based solely on our frame of reference. The first step, as taken here, is being conscious that such differences exist in the first place. For future work, it will be important to quantify just how divergent beliefs are and link this with existing work (such as in-built cognitive biases and preferences) on how organisms may in practice deal with these errors^29,33^. In turn, this may have considerable interesting overlap with the economics literature where behaviour under incorrect models is studied^13–15^. For the particular experiments focused on here, it will be fruitful to consider explicit decision rules and currencies in order to generate *in silico* data. Once done, it will be interesting to see if decisions based on expected rewards, variances or a combination of both most closely resembles the wealth of existing experimental data. Further, to gain a more complete understanding of risk motivations, it will be important to extend this type of analysis to the large class of risk in delay experiments (as opposed to simply risk in reward amount experiments)^18^.
